# Recent ecological transitions in China: greening, browning, and influential factors

**DOI:** 10.1038/srep08732

**Published:** 2015-03-04

**Authors:** Yihe Lü, Liwei Zhang, Xiaoming Feng, Yuan Zeng, Bojie Fu, Xueling Yao, Junran Li, Bingfang Wu

**Affiliations:** 1 State Key Laboratory of Urban and Regional Ecology, Research Center for Eco-Environmental Sciences, Chinese Academy of Sciences, Beijing 100085, China; 2 Joint Center for Global Change Studies, Beijing 100875, China; 3 Institute of Remote Sensing and Digital Earth, Chinese Academy of Sciences, Beijing 100101, China; 4 Institute of Desertification Studies, Chinese Academy of Forestry, Beijing 100091, China; 5 Department of Geosciences, University of Tulsa, 800 S. Tucker Drive, Tulsa, USA

## Abstract

Ecological conservation and restoration are necessary to mitigate environmental degradation problems. China has taken great efforts in such actions. To understand the ecological transition during 2000–2010 in China, this study analysed trends in vegetation change using remote sensing and linear regression. Climate and socioeconomic factors were included to screen the driving forces for vegetation change using correlation or comparative analyses. Our results indicated that China experienced both vegetation greening (restoration) and browning (degradation) with great spatial heterogeneity. Socioeconomic factors, such as human populations and economic production, were the most significant factors for vegetation change. Nature reserves have contributed slightly to the deceleration of vegetation browning and the promotion of greening; however, a large-scale conservation approach beyond nature reserves was more effective. The effectiveness of the Three-North Shelter Forest Program lay between the two above approaches. The findings of this study highlighted that vegetation trend detection is a practical approach for large-scale ecological transition assessments, which can inform decision-making that promotes vegetation greening via proper socioeconomic development and ecosystem management.

The global human population has been continuously increasing, and 2 to 4 billion additional people will live on our planet by 2050^1^,^2^. Human activities have thus far imposed large ecological footprints on the earth. Specifically, landscapes transformed by agriculture and human settlements cover three-fourths of the global ice-free land^3^. Ecological and environmental degradation caused by the ever-increasing extent and intensity of human activities have been and will continue to be the most perplexing challenges in reaching global sustainability^4^. Under this challenging context, ecological restoration and conservation have been widely conceived and implemented as important corrective measures against environmental degradation problems facilitating sustainability at various scales^5^,^6^,^7^. Protected areas, which cover approximately 13% of the global land surface, are the dominant form of ecological conservation efforts^8^. However, ecological restoration is usually project-based. Given widely established protected areas and widely implemented ecological restoration programmes, the effectiveness of these conservation and restoration efforts has recently become an attractive research topic with critical management implications^5^,^8^,^9^,^10^. Consequently, there is an increasing need to evaluate quantitatively both the ecological effects of human socioeconomic development and the effectiveness of ecological conservation and restoration, particularly on large spatial scales, to improve decision support for land use and ecological restoration planning and implementation^5^,^11^,^12^,^13^.

Such evaluations have proven to be very challenging due to many factors, such as the lack of baseline and reference data, inadequate planning and monitoring, socioeconomic complexity, and spatiotemporal uncertainty^14^,^15^. Fortunately, remote sensing data with the advantages of broad spatial coverage and high temporal resolution have been widely used in impact assessments of land use change and ecological restoration^16^,^17^,^18^. In this respect, the vegetation characteristics measured by the Normalized Difference Vegetation Index (NDVI) derived from Moderate Resolution Imaging Spectroradiometer (MODIS), or coarser remote sensing imageries, have been frequently used at large spatial scales^19^,^20^. However, interferences introduced by the spectral characteristics of soil components can result in uncertainties in the NDVI products^21^. Thus, the linear spectral unmixing method has been proposed to tackle this problem to estimate the fractional vegetation cover (FVC) on the earth's surface^22^. FVC is a key biophysical parameter for studying the status and functioning of terrestrial ecosystems across spatiotemporal scales^23^.

As the largest developing nation, China has one-fifth of the world's population. The country's socioeconomic status continues to grow rapidly and it suffers from severe ecological degradation problems^24^. The estimated economic costs of the interrelated problems associated with ecological degradation (e.g., resource depletion, environmental pollution, and ecological degradation) have amounted to over 13% of the national gross domestic product^25^. Widespread ecological degradation has raised serious concerns from both the Chinese government and the general public. As a result, the Chinese government has launched several large-scale ecological rehabilitation and conservation programmes since the late 1990s, such as the Natural Forest Protection Program (NFPP)^24^ and the Three-North Shelter Forest Program (TNSFP)^26^. Furthermore, China has previously established a protected area system, which manages approximately 15% of the country's territory^27^. Due to the effects of multiple degenerative and regenerative forces, it is important to recognise the spatiotemporal characteristics of ecological change in China as a typical case for better decision support and policy making on land use and ecosystem management towards harmonising human-nature relationships in the developing world.

Thus, the objectives of the present study included the following: (1) to quantify the changes in vegetation cover over the last decade (2000–2010) based on the annual maximum FVC for the 11 years, which were represented mainly as greening (restoration) and browning (degradation) at the national and provincial levels in China; (2) to analyse the influential factors in greening and browning trends; and (3) to discuss the policy and practical implications of vegetation change analyses. Based on these analyses, we can test the following research hypotheses: (1) ecological restoration (greening) and degradation (browning) coexisted at regional scales owing to complex driving forces; (2) ecological transition (vegetation change) in China between 2000 and 2010 was most relevant to socioeconomic factors; and (3) large scale ecological conservation and restoration efforts have been heterogeneous in their performance.

## Results

### Vegetation change trends at the national level

**Figure 1:**
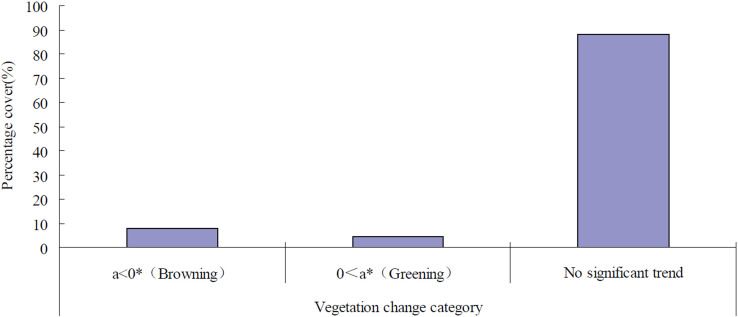
The percentage coverage of vegetation change trends in China from 2000 to 2010. *Statistically significant at the p < 0.05 level.

**Figure 2:**
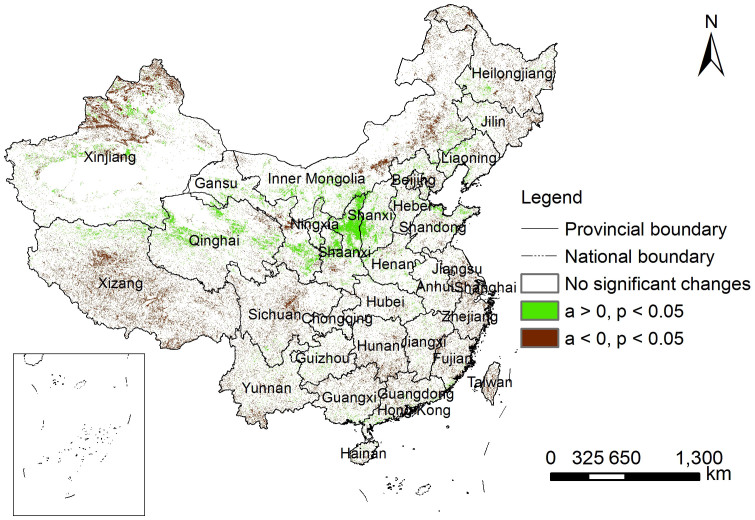
The spatial pattern of vegetation browning (*a* < 0, P < 0.05) and greening (*a* > 0, P < 0.05) at the national scale from 2000 to 2010. The map was created using ESRI ArcGIS 9.3.

Within the last decade, 88.05% of the Chinese territory was detected to have no significant vegetation change; however, the percentage of the coverage of statistically significant vegetation changes was much smaller, 4.28% of the land mass in China for vegetation greening and 7.67% of the land mass for browning (Figure 1). Therefore, most of the landscape in China from 2000 to 2010 had a stable vegetation cover status. Processes of significant vegetation greening and browning coexisted, which resulted in a slight net browning trend at the national level. Spatially, the significant vegetation greening trends were detected mostly in the northern part of China, whereas the significant browning trend in vegetation cover change was scattered across the country with a much more fragmented distribution pattern (Figure 2).

### Vegetation change trends at the provincial level

Significant vegetation change trends of greening and browning, detected simultaneously at the provincial level, showed high spatial variability (Figure 2 and Supplementary Table S1). Eleven out of the 34 provincial administrative regions experienced net significant vegetation greening, whereas the remaining 23 provincial administrative regions experienced significant vegetation browning during the period between 2000 and 2010. The top five provincial administrative regions ranked by a vegetation greening surplus (the percentage of significant vegetation greening subtracted by the percentage of significant vegetation browning) in descending order were Shaanxi, Shanxi, Ningxia, Gansu, and Qinghai. By contrast, the top five provincial administrative regions ranked by a vegetation browning surplus (the percentage of significant vegetation browning subtracted by the percentage of significant vegetation greening) in descending order were Taiwan, Shanghai, Zhejiang, Jiangsu, and Xizang (Tibet).

### Correlations of vegetation change trends with climate and socioeconomic change factors

**Figure 3:**
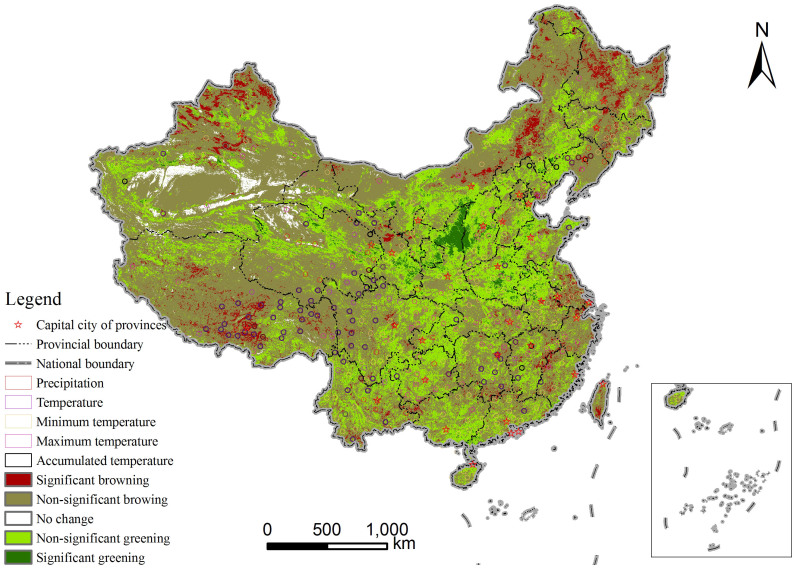
Geographical distribution of the represented areas of the meteorological stations detected to have significant climate variation trends in the 2000s. The map was created using ESRI ArcGIS 9.3.

We performed correlation analysis at the meteorological stations detected to exhibit statistically significant (*p* < 0.05) trends on climate parameters, including annual temperature variables (maximum, minimum, mean, and accumulated temperature above 10 degrees Celsius) and precipitation (Table 1). The percentage of meteorological stations with statistically significant trends were 33.8% (201 stations), 40.8% (267 stations), 17.1% (111 stations), 15.4% (98 stations), and 5.9% (38 stations) of the total number of meteorological stations for annual maximum temperature, annual minimum temperature, annual mean temperature, accumulated temperature over 10 degrees Celsius, and annual precipitation, respectively (Figure 3). The correlation coefficients were calculated between the variation trends of climate parameters and the area percentage of the pixels with significant vegetation change trends (i.e., greening and browning) in the areas surrounding the meteorological stations with significant trends of climate variables within a 20 km radius. Results indicated that climate variations from 2000 to 2010 across China were highly heterogeneous for different variables. This result suggested that significant temperature trends were mainly represented at regional scales, but significant trends in annual precipitation were more restricted to smaller scales. Statistically significant correlations were detected between the aerial percentage of pixels with significant vegetation greening and significant trends in the annual maximum temperature (−0.168), accumulated temperature over 10 degrees Celsius (−0.277), and the aerial percentage of pixels with significant vegetation browning and significant trends in the annual mean temperature (−0.594) and accumulated temperature over 10 degrees Celsius (0.332) (Table 1). All other correlation coefficients were not statistically significant.

To determine the effects of socioeconomic factors on vegetation change in China, we performed correlation analyses between the area ratios of significant vegetation greening (*a* > 0*, p* < 0.05) and vegetation browning (*a* < 0, *p* < 0.05) and changes in socioeconomic variables, including population, economic productivity, investment, employment, income, consumption and expenditures at the provincial level administration regions. Results indicated that seven and six socioeconomic variables correlated significantly with significant trends in vegetation browning and greening, respectively (Table 2). The total human population, the working age population, total employment, and employment in urban areas had significant positive correlations with vegetation browning trends at the provincial level, which implied that heavy human population pressure could be a possible predicator or driving factor for ecological degradation. However, improvements in agricultural productivity (primary industry product), total investments in fixed assets, and the per capita consumption expenditures of rural households might contribute to the mitigation of the vegetation browning problem as the correlation coefficients were negative and significant. For trends in vegetation greening, the six significant socioeconomic factors ranked by the correlation coefficients in descending order were per capita consumption expenditures of rural households > gross domestic product (GDP) = primary industry product = rural household consumption expenditures > household consumption expenditures > urban employment, which implied that general economic development and improvements in human welfare could likely contribute to ecological restoration. Among the above socioeconomic factors, employment in urban areas, primary industry product, and per capita consumption expenditures of rural households represented statistically significant correlations with both vegetation browning and greening trends, which reinforced the possible effects of urbanisation on ecological degradation and the contributions of agricultural productivity and rural welfare to ecological restoration.

### The effects of ecological conservation and restoration efforts

**Figure 4:**
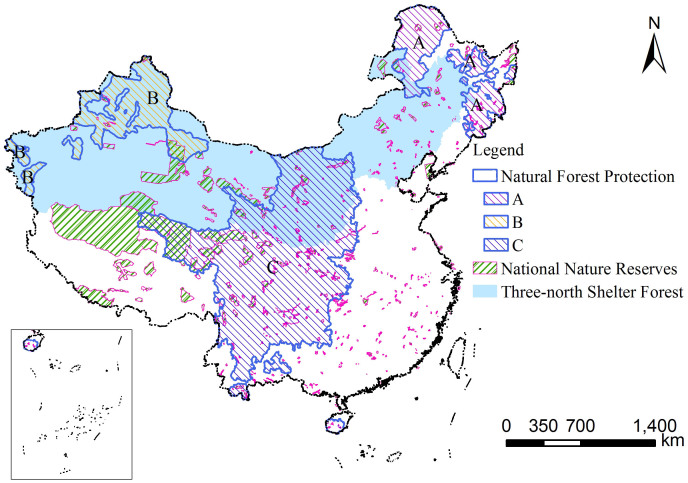
Spatial extent of the three large-scale ecological conservation and restoration programs. (A), (B), and (C) represent the areas under the natural forest protection program in northeast China, northwest China, the upper reaches of the Yangtze River and the upper and middle reaches of the Yellow River. The map was created using ESRI ArcGIS 9.3.

Ecological conservation and restoration are the two main approaches used globally to combat ecological degradation by reducing human use pressures on natural ecosystems or by facilitating ecosystem recovery from degraded situations. China has established a large protected area system beginning in the 1950s^27^ and has implemented large-scale ecological conservation and restoration programs beginning at the end of the 1990s^24^. Both the management of nature reserves and the implementation of large-scale ecological conservation and restoration efforts are expected to elicit significant effects on vegetation change. We used the Chinese National Nature Reserves (NNR), the areas for the Three North Shelter Forest Program (TNSFP), and the areas for the Natural Forest Protection Program (NFPP)^24^, which account for approximately 10%, 40%, and 34% of the Chinese territory, respectively, to detect the effects of ecological conservation and restoration efforts on vegetation change trends (Figure 4). A comparison of the vegetation change trends of these three types of areas with those at the national level revealed that all of the above-mentioned ecological conservation and restoration efforts generally contributed to the mitigation of vegetation browning and the enhancement of vegetation greening (Table 3). Among the three project implementation areas, NNR management showed marginal positive effects on vegetation change as the percentage coverage of different vegetation change categories were highly similar to the national status. NFPP was most effective at facilitating vegetation greening, whereas TNSFP was most effective at curbing vegetation browning. Taking all the vegetation change categories into consideration, the overall comparative effectiveness of the three large scale ecological programs could be ranked as Natural Forest Protection > Three-north Shelter Forest > National Nature Reserves. For the different sub-regions of NFPP, the most effective was in the upper reaches of the Yangtze River and the upper and middle reaches of the Yellow River (C), whereas the areas under NFPP in northeast China (A) and northwest China (B) were not as effective (Figure 4 and Table 3). These results revealed the interesting finding that even under the same policy context of forest protection with incentive measures, the effectiveness of large-scale ecological conservation programs can vary geographically.

## Discussion

### Possible influential factors for vegetation change trends

Climate is generally considered to be an important biophysical factor influencing vegetation growth. However, research findings thus far have been mixed on the significant^28^,^29^ and non-significant^30^,^31^ effects of climate factors on vegetation change. This phenomenon may be explained by the nonlinearity of vegetation responses to climate and the spatiotemporal variations of these vegetation responses^32^,^33^. The present research revealed that climate variations were possibly statistically significant influential factors for decadal vegetation change trends (Table 1). However, the characteristics of climate variation and the interactions between climate and vegetation growth can be highly spatial heterogeneous. It has been revealed that the mean climate variation trend at the national level could be represented as warming and drying in the 2000s, with high spatial heterogeneity and higher change rates than in the past several decades^34^. This research further uncovered a general significant negative influence of significant climate warming on vegetation greening (Table 1). This phenomenon may be due to water stress under such climate warming trends^35^, which can be very important, particularly in the growing season as indicated by the present research. However, warming in non-growing seasons seems to mitigate vegetation browning, which needs to be further verified in future research.

Vegetation change may also be affected by socioeconomic factors, such as land use policies, population, and income^36^,^37^. Correlation analyses between the percentage coverage of significant vegetation browning or greening and the percentage change in socioeconomic variables at the provincial level implied that population pressure could be a general significant influential factor for vegetation browning, which is very common in developing countries. For example, Bangladesh has experienced landscape fragmentation and the loss of landscape structural and functional diversity caused by population growth, economic development, and rapid urban expansion^38^,^39^, South Africa has incurred ecological degradation due to the development of extensive impoverished rural communities^40^, and in southern Brazil, the significant contributions of age structure and population density are the most important driving factor for ecological degradation^41^. However, regional economic vitality in general and agriculture vitality in particular, and improvements in the economic welfare and quality of life, especially in rural areas, could potentially exert significant positive effects on vegetation greening (Table 2). Empirical studies and case analyses verified the existence of the positive effects of agricultural improvements^42^ and the transition of rural socio-ecological systems promoting lower human pressure and higher human welfare in rural areas^43^ on vegetation greening and ecological restoration in China. One important force for positive socioeconomic transitions is the rural-urban migration of rural labour forces with the development of the market economy, which has been shown to facilitate both ecological restoration and human welfare in rural China^43^,^44^,^45^. Internationally, integrative analyses at the national scale in Italy and Nigeria also suggested possible contributions of economic productivity on ecological restoration and the mitigation of land degradation^46^,^47^. Therefore, improvements in economic welfare and the quality of life in rural areas may be beneficial for ecological restoration, which can be promoted by highly efficient agricultural industries and sustainable urbanisation practices that induce population migration from rural to urban areas. However, resource-saving approaches in urbanisation, production, and consumption are necessary to realise positive transitions facilitating ecological restoration.

### Policy and practical implications of vegetation change analyses

The FVC used in the present study was better than the NDVI because it considered interference by non-vegetation covered areas^48^. On the basis of the FVC, large-scale vegetation change trends, which were represented as greening and browning, can be efficiently detected. Thus, vegetation greening and browning can be used as simple and cost-effective indicators for performance assessments of large-scale ecological restoration and conservation efforts. The effective implementation of such efforts has also been proved to be the dominant factor facilitating vegetation greening trends at regional scales^49^,^50^,^51^. Both biophysical and socioeconomic factors can have impacts on vegetation change, but the present study indicated that socioeconomic factors were more significant in explaining both greening and browning vegetation change trends. Human populations also exerted significant pressures on ecosystems as shown by their ecological footprint of 1.5 times our planet earth in 2008, and such pressures been increasing over the last several decades^52^. Our analysis revealed the negative effects of an increase in human population on vegetation greening (Table 2). Alleviating human pressures on ecosystems is inevitably a difficult task for both ecological conservation and restoration initiatives. This study suggests potential socioeconomic approaches to facilitate ecological restoration and conservation, including urbanisation, advancements in agricultural productivity, human welfare improvements, especially in rural areas, and investments in payments for ecosystem services (Table 2).

Taken together, the findings of this study highlighted the potential wide usability of vegetation fraction trend detection as an important approach with high cost efficiency in evaluating the effectiveness of large-scale ecological conservation and restoration efforts. Conservation efforts beyond nature reserve boundaries deserve to be advocated for their practical effectiveness. Attention must be paid to spatial heterogeneity in both the planning and implementation of such large-scale ecological conservation efforts. Policies also need to be prepared for the adaptation of ecological conservation and restoration efforts to climate warming challenges, especially in the growing season. Furthermore, properly directed socioeconomic forces can help to reduce vegetation browning and promote vegetation greening. The present study supported the three research hypotheses that ecological restoration (greening) and degradation (browning) coexisted at regional scales, ecological transitions (vegetation change) in China from 2000 to 2010 were most related to socioeconomic factors, and the performance of large-scale ecological conservation and restoration efforts has been heterogeneous.

## Methods

### Data sources

NDVI products derived from MODIS imagery with a ground resolution of 250 m from 2000 to 2010 with a 16-day time interval were used to estimate the vegetation fraction of ground cover. Daily precipitation and temperature data from over 600 meteorological stations across China from 2000 to 2010 were retrieved from the China Meteorological Data Sharing Service System. Land use maps in China (2000 and 2010) at 30 m ground resolution^53^ were used as supplementary data for vegetation fraction estimation. Socioeconomic data spanning 2000–2010 at the provincial level from the China Statistical Yearbooks^54^,^55^ were collected to analyse the influencing factors underlying vegetation change. Selected provincial-level socioeconomic development variables were related to human population, economic productivity, investment, employment, income, consumption and expenditures.

### Vegetation change and climate trend estimation

Fractional Vegetation Cover (FVC) is a comprehensive quantitative indicator to describe the condition of vegetation. A dimidiate pixel model, which is a simplified linear spectral unmixing method, was used to calculate FVC^33^. An assumption of the model is that a pixel contains only two elements of vegetation and soil, which can be represented as follows:
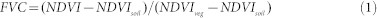
Where NDVI is the Savitzky-Golay filtered results of MODIS-NDVI products with 250 m spatial resolution. *NDVI_soil_* and *NDVI_veg_* are two key input parameters, which represent the NDVI value of pure pixels of bare soil and vegetation, respectively. The selections of *NDVI_soil_* and *NDVI_veg_* mainly depend on the China land cover map, high spatial resolution data, hyperspectral images and field samples. For the different types of vegetation, *NDVI_veg_* is the mean value of all pure vegetation pixels selected from high spatial resolution data in the typical study area. *NDVI_soil_* is calculated based on the pure soil pixels, which are detected from the hyperspectral image compared with field spectral measurements. The detailed process of FVC estimation can be found in relevant literature^56^,^57^.

Due to the phenology of vegetation growth, *FVC* is dynamic in the growing season. Thus, the annual maximum *FVC* was used to represent the optimal annual status of vegetation cover. For each pixel, we used simple linear fitting to quantify the multi-annual vegetation change trends. We also estimated the trend of climate variables (annual maximum, minimum, and mean temperatures; accumulated temperature above 10 degrees Celsius; and annual precipitation) using linear regression at the meteorological stations. The linear regression approach has been widely used in trend detection for vegetation change and climate variations^58^,^59^. The least-squares regression equation can be represented as follows:
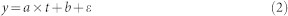
In equation (2), y is the dependent variable of the annual maximum *FVC* or climate variables in year t (t ranges from 2000 to 2010, t∈N); therefore, the sample size of the annual maximum *FVC* (or climate variables) for each pixel (or meteorological station) in the vegetation change (or climate variation) trend estimation is 11; *a* is the slope of the equation (or change rate of the linear trend) and *b* is the intercept of the equation; *t* is the year; and εis the random error. When *a* < 0, the pixel experienced a vegetation change trend of browning, which represents a degradation in the vegetation cover. By contrast, when *a* > 0, the pixel experienced a vegetation change trend of greening, representing a regeneration or restoration of vegetation cover. For climate variables, variation trends can be expected if a≠0. The trends are taken as statistically significant at the p < 0.05 level. Equation (2) was solved using the MATLAB REGRESS function with an orthogonal least squares method^60^. The syntax is [b, bint, r, rint, stats] = regress (y, X), which returns a 1-by-4 vector that contains, in order, the R^2^ statistic, the F statistic and its p value, and an estimate of the error variance.

### Correlation analysis

Pearson's correlation analyses were performed using the function of [r, p] = corr (X, Y) in MATLAB Version R2010b, which can return both the correlation coefficients and p-value (significant test) simultaneously, where r and p represent the correlation coefficient matrix and the matrix of significant test results and X and Y are the respective vectors of vegetation change and climate variation (or socioeconomic change) characteristics.

The climate variables considered in the correlation analysis include annual maximum temperature, annual minimum temperature, annual mean temperature, accumulated temperature over 10 degrees Celsius, and annual precipitation. For each meteorological station, the variation trends of the above five variables are detected using equation (2). After this step, the meteorological stations with statistically significant variation trends for the five climate variables are determined. For each meteorological station with significant trends of climate variation, a circular buffer area with a radius of 20 km (i.e., the representative area covering approximately 1256.6 km^2^) was created using the relevant analysis tool in the ARCGIS 9.3 environment. Then, the area percentage of the pixels with statistically significant vegetation change trends (i.e., greening and browning) is calculated in the buffer area. Finally, each pair of climate variable trends (a_climate_, *p* < 0.05) at each meteorological station and the area percentage of vegetation change trends of significant greening and browning in the buffer area for each station were used in correlation analyses.

Correlation analyses between vegetation change and socioeconomic variables were based on the percentage change in socioeconomic variables in 2010 compared with 2000 
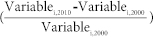
, as well as the percentage coverage of the areas with statistically significant vegetation browning (*a* < 0 and *p* < 0.05) and greening (*a* > 0 and *p* < 0.05) trends from 2000 to 2010 at the provincial level (31 provincial administrative regions from mainland China). Twenty socioeconomic variables from four categories, including human population, general economic vitality, income, and consumption, were considered in the correlation analysis (Table 4).

### Comparative effectiveness analysis on ecological conservation and restoration efforts

The national status of vegetation change categories (significant greening, browning, and no change) is used as the reference to estimate the comparative effectiveness of large-scale ecological conservation and restoration efforts on curbing vegetation degradation (browning) and promoting vegetation restoration (greening). The total comparative effectiveness of ecological conservation or restoration efforts can be calculated as *e_i_* = *e_i_*_1_ + *e_i_*_3_ − *e_i_*_2_, where *e_ij_* = *P_ij_*/*N_j_* [j = 1(greening), 2 (browning), and 3(no change)], within which *N_j_* and *P_ij_* are the area percentage of the above three vegetation change trends at the national level and in different ecological conservation and restoration regions, respectively. The conservation and restoration effectiveness is higher if the *e_i1_* and *e_i3_* values are bigger and the e_i2_ value is smaller. The total comparative effectiveness of ecological conservation and restoration efforts can be ranked using *e_i_*.

## Author Contributions

B.F. and Y.L. designed the study. L.Z., X.F., Y.Z., X.Y. and B.W. performed data collection and analysis. Y.L. interpreted the results and analysed the data. J.L. revised the early version.

## Supplementary Material

Supplementary Information Supplementary Table S1

## Figures and Tables

**Table 1:** Correlation between climate variation trends and the percentage cover of the pixels with significant vegetation change trends in the circling area with a 20 km radius surrounding the meteorological stations with statistically significant trends

Change trends	Climate variable trends	Annual max T	Annual Min T	Annual Mean T	Accumulated T ≥ 10°C	Annual Precipitation
Significant greening (%)	Correlation coefficients	−0.168*	−0.048	0.114	−0.277**	0.209
Significant browning (%)		0.096	−0.037	−0.594**	0.332**	−0.278
Number of meteorological stations with significant trends*		201	267	111	98	38
Total number of meteorological stations		595	655	649	637	649

T = temperature;

*P < 0.05;

**P < 0.01.

**Table 2:** Correlations between vegetation change area percentages and socioeconomic factors

	Area ratio of different vegetation change trends	
Change from 2000–2010	Browning (*a* < 0, p < 0.05)	Greening (0 < *a*, p < 0.05)
Total population	0.53**	
Working age population (15–64)	0.48**	
Total employment	0.50**	
Urban employment	0.68***	−0.41*
Gross Domestic Product		0.42*
First industry product	−0.68***	0.42*
Invest	−0.44*	
Household consumption expenditures		0.37*
Rural household consumption expenditures		0.42*
Per capita consumption Expenditures of rural households	−0.50**	0.52**

*Significant at p < 0.05;

**Significant at p < 0.01;

***Significant at p < 0.001.

**Table 3:** Comparative effectiveness of different large-scale ecological conservation and restoration projects

	Greening (1)	Browning (2)	No change (3)	Total effectiveness (*e_i_*)
Natural Forest Protection (1)	1.947	0.760	0.975	1.187
Natural Forest Protection (1-A)	0.091	1.366	1.012	−1.275
Natural Forest Protection (1-B)	0.681	1.844	0.942	−1.163
Natural Forest Protection (1-C)	1.937	0.766	0.975	1.171
Three-north Shelter Forest (2)	1.576	0.731	0.995	0.845
National Nature Reserves (3)	1.043	0.928	1.004	0.118

**Table 4:** The socioeconomic indicators used in the correlation analyses

Category	Indicators
Human population	Total population; working age population (15–64); population with a college education and above; total employment; employment in urban areas; employment in rural areas
General economic vitality	Gross Domestic Product; primary industry product; secondary industry product; tertiary industry product
Income	Government revenue; per capita annual net income of rural households; per capita annual disposable income of urban households
Consumption	Final consumption expenditures; final consumption expenditures by urban households; final consumption expenditures by rural households; per capita consumption expenditures of rural households; per capita consumption expenditures of urban households; government expenditures; total investment in fixed assets
