# Supplementary material for: Recent ecological transitions in China: greening, browning, and influential factors
Source: Sci Rep. 2015 Mar 4;5:8732. doi: 10.1038/srep08732 (PMC4348646; doi:10.1038/srep08732)
Supplement: Supplementary Information — Supplementary Table S1 [file srep08732-s1.doc]

**Recent ecological transitions in China: greening, browning, and influential factors**

Yihe Lü^1^, Liwei Zhang^1^, Xiaoming Feng^1^, Yuan Zeng^2^, Bojie Fu^1*^, Xueling Yao^3^, Junran Li^4^, Bingfang Wu^2^

**Author affiliations:**

1. State Key Laboratory of Urban and Regional Ecology, Research Center for Eco-Environmental Sciences, Chinese Academy of Sciences, Beijing 100085, China.

2. Institute of Remote Sensing and Digital Earth, Chinese Academy of Sciences, Beijing 100101, China.

3. Institute of Desertification Studies, Chinese Academy of Forestry, Beijing 100091, China.

4. Department of Geosciences, University of Tulsa, 800 S. Tucker Drive, Tulsa, USA.

***Corresponding author:**

State Key Laboratory of Urban and Regional Ecology, Research Center for Eco-Environmental Sciences, Chinese Academy of Sciences, PO Box 2871, Beijing 100085, China

Email: bfu@rcees.ac.cn; Tel.: 86-10-62923557; fax: 86-10-62923557

Supplementary Table S1.

The area percentage of vegetation change categories at the provincial level in China during 2000 and 2010.

| Provincial Level  Administrative Regions | Percentage cover by the vegetation change rate (*a*) category (%) | | | | |
| --- | --- | --- | --- | --- | --- |
|  | No significant changes | *a<0, p<0.05* | *a>0, p<0.05* | Greening surplus | Browning surplus |
| Beijing | 84.5 | 12.2 | 3.3 |  | 8.9 |
| Tianjin | 84.1 | 8.1 | 7.8 |  | 0.3 |
| Hebei | 88.6 | 5.6 | 5.8 | 0.2 |  |
| Shanxi | 80.2 | 2.1 | 17.8 | 15.7 |  |
| Liaoning | 88.9 | 4.3 | 6.8 | 2.5 |  |
| Jilin | 89.3 | 8.5 | 2.2 |  | 6.3 |
| Heilongjiang | 89.3 | 8.9 | 1.9 |  | 7 |
| Shanghai | 79.8 | 19.1 | 1.1 |  | 18 |
| Jiangsu | 85.6 | 13.5 | 1 |  | 12.5 |
| Zhejiang | 85.1 | 14.3 | 0.6 |  | 13.7 |
| Anhui | 92.1 | 4.9 | 3 |  | 1.9 |
| Fujian | 85.2 | 13 | 1.9 |  | 11.1 |
| Jiangxi | 89.3 | 8.2 | 2.5 |  | 5.7 |
| Shandong | 88.1 | 5.1 | 6.8 | 1.7 |  |
| Henan | 91.3 | 2.9 | 5.8 | 2.9 |  |
| Hubei | 92.5 | 5.1 | 2.4 |  | 2.7 |
| Hunan | 88.1 | 11 | 0.9 |  | 10.1 |
| Guangdong | 86.6 | 9.7 | 3.7 |  | 6 |
| Guangxi | 88.8 | 8.8 | 2.3 |  | 6.5 |
| Hainan | 89.7 | 7.3 | 3 |  | 4.3 |
| Chongqing | 92.2 | 6.1 | 1.7 |  | 4.4 |
| Sichuan | 89 | 9.9 | 1.2 |  | 8.7 |
| Guizhou | 91.7 | 4.5 | 3.8 |  | 0.7 |
| Yunnan | 88.9 | 8.9 | 2.2 |  | 6.7 |
| Xizang | 85.1 | 13.3 | 1.7 |  | 11.6 |
| Shaanxi | 69.1 | 3.7 | 27.3 | 23.6 |  |
| Gansu | 86.5 | 3.9 | 9.6 | 5.7 |  |
| Qinghai | 88.3 | 3.4 | 8.3 | 4.9 |  |
| Ningxia | 85.5 | 2.9 | 11.6 | 8.7 |  |
| Xinjiang | 90.1 | 7 | 2.9 |  | 4.1 |
| Taiwan | 80.1 | 19.6 | 0.3 |  | 19.3 |
| Hong Kong | 92.7 | 3 | 4.3 | 1.3 |  |
| Macau | 95.1 | 2.2 | 2.7 | 0.5 |  |
| Inner Mongolia | 90.5 | 6 | 3.5 |  | 2.5 |
